# Laser auriculotherapy for the treatment of pain: a scoping review

**DOI:** 10.1007/s10103-026-05000-5

**Published:** 2026-09-14

**Authors:** Filipe José Pereira Magalhães, Jéssica Pinheiro Carnaúba, Odaleia de Oliveira Farias, Bernardo Diniz Coutinho, Marli Teresinha Gimeniz Galvão, Mariana Lima Vale

**Affiliations:** 1 Postgraduate Program in Pharmacology, Universidade Federal do Ceará, Fortaleza, Brazil; 2 Northeast Network for Training in Family Health (RENASF), Universidade Federal do Ceará, Fortaleza, Brazil; 3 Postgraduate Program in Nursing, Universidade Federal do Ceará, Fortaleza, Brazil; 4 Department of Physical Therapy, Universidade Federal do Ceará, Fortaleza, Brazil

**Keywords:** Auriculotherapy, Pain, Laser, Photobiomodulation

## Abstract

Purpose: While auriculotherapy is utilized for pain management, the specific application of photobiomodulation (PBM) within this modality remains poorly characterized. This scoping review aims to map the available evidence on PBM in auriculotherapy for pain relief, specifically characterizing therapeutic protocols and laser parameters to guide future research. Methods: Following Joanna Briggs Institute methodology, major databases were searched for clinical trials evaluating auricular PBM for pain. The methodological quality of the included studies was assessed using the PEDro scale. This review included studies focused on clinical trials of auricular PBM for pain management, while excluding literature reviews, observational designs, protocols, and case studies. The final search was conducted in May 2025. Results: Seven studies were included. PBM in auriculotherapy demonstrated positive trends in pain reduction for chronic lower back pain and temporomandibular dysfunction. However, no significant differences were found in acute postoperative pain or knee osteoarthritis. Protocols utilized infrared (808–904 nm) or red (650–660 nm) wavelengths, with energy densities ranging from 0.54 to 4 J/cm². The most frequently stimulated points were Shen Men (85.7%), Sympathetic, Kidney, Liver, and Subcortex (42.8% each). Treatment regimens typically consisted of 8 to 10 sessions, performed once or twice weekly. Heterogeneity and incomplete reporting of dosimetry parameters were observed across the literature. Conclusion: Laser auriculotherapy is a promising non-invasive approach for chronic musculoskeletal pain. However, substantial variability in parameters and methodological inconsistencies limit comparability. Standardized reporting and larger controlled trials are essential to define optimal protocols for auricular PBM.

**Supplementary Information:**

The online version contains supplementary material available at https://doi.org/10.1007/s10103-026-05000-5.

## Introduction

Auriculotherapy is a method of diagnosing and treating physical and psychosomatic dysfunctions through the stimulation of specific points on the ear, and is a technique that originated in Traditional Chinese Medicine (TCM) [1]. In this context, a systematic review and meta-analysis that evaluated the effects of auriculotherapy in pain treatment, significant reductions were observed in pain scores and analgesic consumption across postoperative, acute, and chronic pain conditions [2]. Futhermore, specifically the studies investigating the use of auriculotherapy for the treatment of low back pain demonstrated promising results in reducing chronic pain intensity [3]. Considering the neuroanatomical characteristics of the auricle, transcutaneous electric stimulation can generate neuromodulatory effects by activating the complex innervation of the ear, which includes the great auricular nerve, auriculotemporal nerve, lesser occipital nerve, and especially the auricular branch of the vagus nerve (ABNV) [4–5].

Furthermore, along with auriculotherapy, photobiomodulation (PBM) has been used. This technique is based on the use of lasers (or LED light) that produce low-intensity light, a non-invasive method that has shown effectiveness in reducing inflammation and relieving pain by stimulating auricular acupuncture points [6]. The main advantage of the laser auriculotherapy technique is that it does not require the use of needles, making the process less invasive and painless. Regarding its mechanism, laser increases nerve impulses by raising the excitability of free nerve endings, mainly through stimulating the Na⁺/K⁺ pump in cell membranes. PBM triggers significant cellular responses through photon absorption by mitochondrial chromophores, primarily Cytochrome C Oxidase. This interaction boosts mitochondrial activity, leading to an increase in adenosine triphosphate (ATP) and reactive oxygen species (ROS) production. Furthermore, PBM modulates intracellular calcium levels, protein synthesis, and enzymatic activity. Combined, these biochemical changes may enhance neural activity and impulse conduction [7–8].

**Fig. 1:**
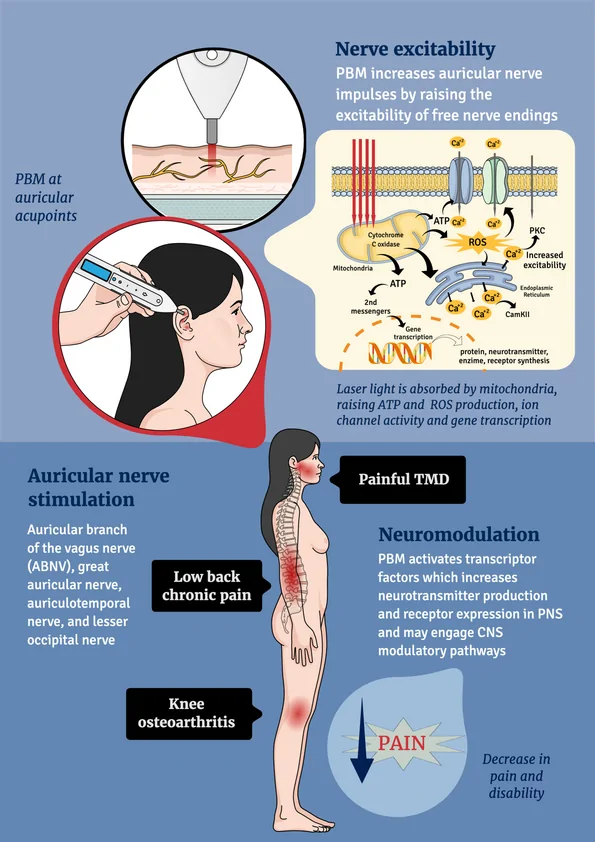
Proposed mechanisms of photobiomodulation (PBM) applied to auricular points for pain management

Thus, by acting directly on neural tissue, laser photobiomodulation may activate similar neuromodulatory mechanisms as auriculotherapy. It may influence auricular nerve structures such as the ABNV, promoting anti-inflammatory effects, pain modulation, and emotional and physiological regulation. Figure 1 illustrates a hypothetical mechanism regarding the effects of PBM on the auricle and its potential impact on the types of pain addressed in this review [Fig. 1]. This convergence supports laser auriculotherapy as a viable method for targeted neuromodulation and therapeutic outcomes comparable to traditional auricular stimulation.

This figure illustrates a proposed and illustrative model of the potential neuromodulatory effects of laser auriculotherapy. PBM may stimulate peripheral nerve endings in the auricular region, activating neural pathways of the Peripheral Nervous System (PNS) that could modulate pain processing within the Central Nervous System (CNS). At the cellular level, photobiomodulation is suggested to enhance mitochondrial function through activation of cytochrome c oxidase, leading to increased production of adenosine triphosphate (ATP) and reactive oxygen species (ROS). ATP may activate purinergic receptors, while ROS may stimulate calcium (Ca²⁺) channels and promote intracellular Ca²⁺ influx. In addition, ROS may induce Ca²⁺ release from the endoplasmic reticulum. The resulting increase in intracellular Ca²⁺ may activate intracellular signaling pathways, including Protein Kinase C (PKC) and Calcium/Calmodulin-Dependent Protein Kinase II (CaMK II). Collectively, these putative molecular and neural mechanisms may contribute to neuronal modulation and help explain the antinociceptive effects reported in studies of laser auriculotherapy [7–8].

It is important to note that, although existing reviews support the effectiveness of auriculotherapy for pain management [2–3], they often do not address PBM as a specific technique within this modality. Given the recognized benefits of auricular stimulation in pain control, there is a clear need to explore the application of photobiomodulation in the auricular region as a potential alternative treatment approach. Furthermore, PBM is therefore employed in both body and auricular acupuncture. Although several reviews have examined its application in conventional laser acupuncture [9–11], no review to date has specifically investigated its use in laser auriculotherapy.

This study aims to map the available evidence on the use of photobiomodulation in auriculotherapy, with a focus on pain relief, treatment outcomes, therapeutic protocols employed and specific laser parameters utilized. Such information is crucial for guiding future clinical and preclinical research involving photobiomodulation as a method of auricular stimulation for pain relief.

## Method

### Study design

This is a scoping review, according to the Joanna Briggs Institute (JBI) method for Scoping Review [12]. The protocol for this scoping review was registered on the Open Science Framework (OSF) platform: https://doi.org/10.17605/OSF.IO/F6WMQ. The database search took place between September 2023 and May 2025.

### Research question

The research question: What evidence of effectiveness is available in the literature regarding the application of laser in auriculotherapy for the treatment of pain? It was developed based on the mnemonic PCC (Population, Concept and Context), with the Population: People with pain; the Concept: Use of laser therapy; and the Context: Auriculotherapy [12].

### Selection criteria

To answer the research question, studies of controlled clinical trials, randomized or not, pilot and feasibility studies, which are published in indexed journals, were considered. Studies available in full, without language or publication date restrictions, were considered. Exclusion criteria: review articles, methodological and observational studies, study protocols, case studies.

According to the JBI method, the following steps were followed: (1) identification of the research question and object; (2) identification of relevant studies; (3) study selection, according to pre-defined criteria; (4) data mapping; (5) summarization of results; (6) presentation of results, identifying the implications for policy, practice or research [12].

### Search strategies

Subsequently, a comprehensive search was performed in the following databases: Medline via PubMed, Scopus, Embase, Latin American and Caribbean Health Sciences Literature (LILACS), Web of Science (WOS), and Cochrane, using all the keywords identified in the initial step.

A consultation was conducted using Medical Subject Headings (MeSH) across all databases. Relevant descriptors and their synonyms were identified using the Boolean operators AND and OR. The search strategies, including the descriptors and their combinations with Boolean operators, are presented in Table 1, and the detailed, platform-specific search syntax for each database is provided in Supplementary Material. 

**Table 1:** Search strategy for identifying studies after consultation

	Population	Concept	Context
Extraction	People with pain	Use of laser therapy	Auriculotherapy
Conversion	Pain	Laser Therapy	Auriculotherapy
Combination	“Pain”; “Ache”; “Physical Suffering”;	“Laser Therapy”; “Low Level Laser Therapy”; “Photobiomodulation”; “Phototherapy”; “Laser”;	“Auriculotherapy”; “Ear Acupuncture”; “Auricular Acupuncture”;
Construction	(“Pain” OR Ache OR “Physical Suffering” )	(“Laser Therapy” OR “Low Level Laser Therapy” OR “Photobiomodulation” OR “Phototherapy” OR “Laser”)	(Auriculotherapy OR “Ear Acupuncture” OR “Auricular Acupuncture”)
Use	((“Pain” OR Ache OR “Physical Suffering”) AND (“Laser Therapy” OR “Low Level Laser Therapy” OR Photobiomodulation OR Phototherapy OR Laser) AND (Auriculotherapy OR “Ear Acupuncture” OR “Auricular Acupuncture”))		

### Screening

During the study selection process, studies were identified, grouped, and uploaded to the Rayyan application [13], where duplicates were removed. Titles and abstracts were then screened based on the inclusion criteria. Full texts of potentially relevant studies were retrieved and assessed in detail. Two independent researchers conducted the selection, and any disagreements at any stage were resolved through discussion.

The research was conducted using the Preferred Reporting Items for Systematic reviews and Meta-Analyses extension for Scoping Reviews, PRISMA-ScR Checklist (Suplementary file) [14].

### Data extraction

To extract the main data, a data collection tool was developed, which was pilot-tested and subsequently refined to better align with the study objectives. The information was compiled into a Microsoft Excel spreadsheet and summarized in tables, presenting the data related to laser parameters, including laser type, output power, wavelength, energy density, other technical specifications reported in the studies and also the acupuncture auricular points used, number of sessions and techniques that were combined with or compared to laser auriculotherapy.

The extracted data were synthesized and presented in tables, and results were reported narratively, accompanied by a discussion of the review findings analyzed in accordance with the objective of this study.

### Methodological quality and risk of bias assessment

The methodological quality of the included studies was independently assessed by two reviewers using the Physiotherapy Evidence Database (PEDro) scale [15]. To ensure consistency and accuracy, all items were cross-checked, and disagreements regarding individual scores were resolved by consensus. This scale evaluates internal validity based on 11 criteria [15]. While total PEDro scores are commonly categorized as ‘poor’ (0–3), ‘fair’ (4–5), ‘good’ (6–8), and ‘excellent’ (9–10), these classifications lack formal psychometric validation [15]. Furthermore, a total score of 8/10 is considered optimal for high-quality trials [15]. Detailed assessment of all 11 criteria for each study is presented in the Supplementary Material, while the synthesis of quality findings is integrated into the Results and Discussion sections.

### Use of AI-assisted tools

Artificial intelligence–assisted tools (ChatGPT, OpenAI) were used exclusively for English language editing and improvement of clarity and grammar. The tool did not generate scientific content, interpret data, perform analyses, or influence the study design, results, or conclusions. All content was reviewed, edited, and approved by the authors, who take full responsibility for the manuscript.

## Results

### Search results

**Fig. 2:**
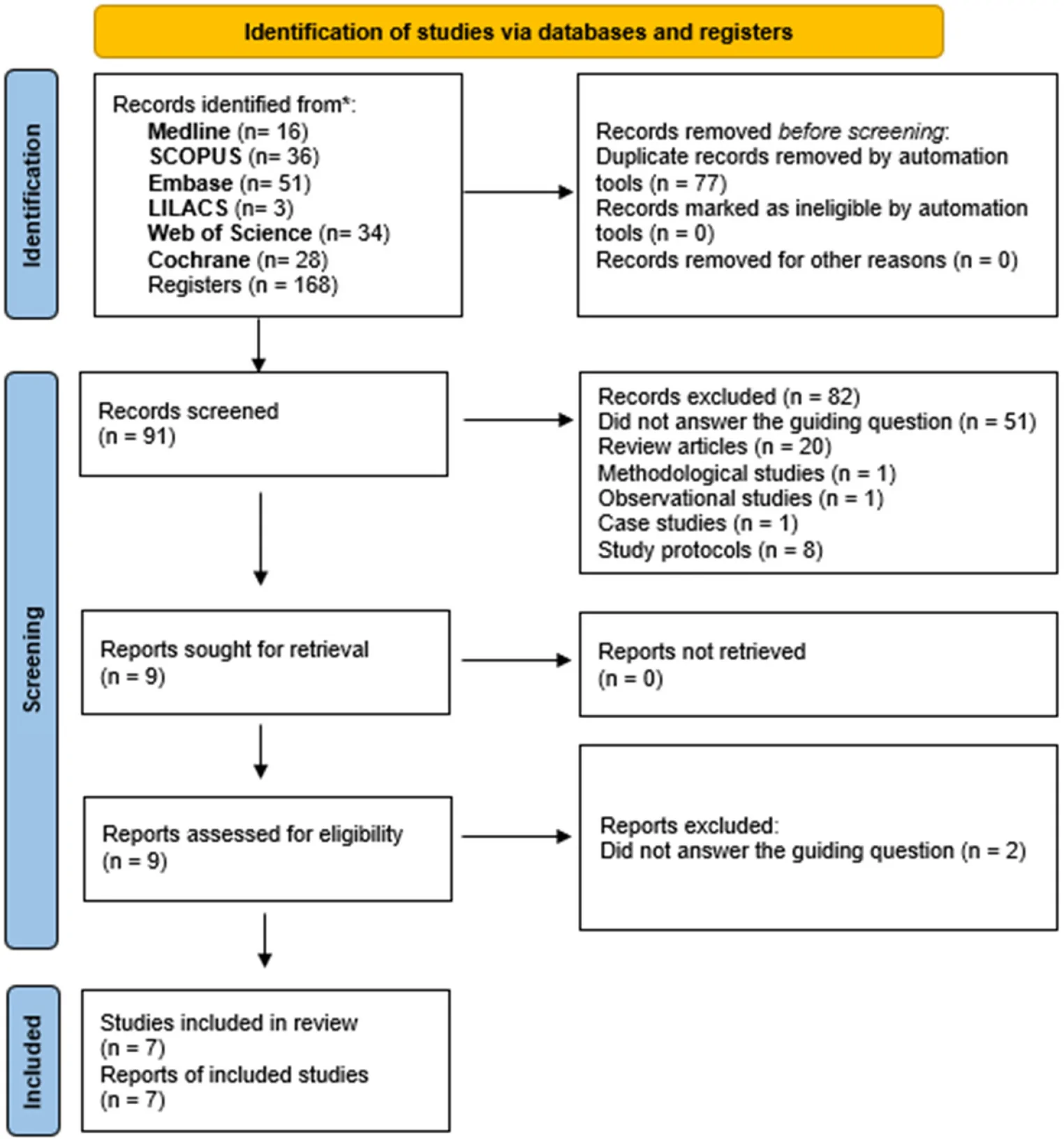
PRISMA 2020 flow diagram for updated systematic reviews which included searches of databases and registers only [16]

A total of 168 records were identified across the databases searched. After application of the eligibility criteria, seven studies were included in the scoping review. The study selection process and final number of included articles are presented in the PRISMA 2020 flow diagram [16] (Fig. 2).

### Outcomes analysis

#### Chronic pain

Discrepancies have been noted among randomized clinical trials evaluating treatments for chronic pain, particularly in cases involving orofacial pain associated with temporomandibular dysfunction (TMD). In the study by Rodrigues et al., laser auriculotherapy was directly compared with an established active treatment—occlusal splint therapy [17]. This randomized, blinded clinical trial included 40 primary school teachers (aged ≥ 18 years) diagnosed with myofascial TMD according to the Research Diagnostic Criteria for Temporomandibular Disorders (RDC/TMD). The study utilized one-way analysis of variance (ANOVA) with Tukey's post-test for quantitative variables and Kruskal–Wallis with Dunn’s post-test for ordinal variables (p < 0.05) to assess pain, disability, and depression levels across three evaluation times (baseline, 4, and 8 weeks) [17]. Both groups demonstrated significant reductions in pain levels, suggesting that laser auriculotherapy may be as effective as occlusal splints in managing TMD symptoms, and that combining the two approaches could further enhance outcomes. An important methodological rigor of this study was the application of strict inclusion criteria: participants were required to report orofacial pain based on a numerical rating scale and undergo clinical assessment using joint and muscle palpation with a dynamometer [17]. Regarding methodological quality, the study achieved a PEDro score of 5/10, which is classified as ‘fair’ quality. While the application of strict inclusion criteria demonstrates a notable level of methodological rigor, the absence of adequate allocation concealment and intention-to-treat analysis represents significant methodological vulnerabilities that should be considered when interpreting the potential for bias in these findings.

In contrast, Fernandes et al. compared laser auriculotherapy to a control group that received no treatment [18]. This study involved 40 participants aged 20 to 45 years with clinical symptoms of TMD, divided into an auriculotherapy group (n = 20) and a control group (n = 20). Data analysis was performed using ANOVA for sleep disorders and non-parametric tests, specifically Mann-Whitney U for between-group comparisons and Wilcoxon for temporal assessments, for anxiety and TMD data [18]. No statistically significant difference in pain levels was found between the two groups (p > 0.05). The authors attributed the lack of efficacy to methodological limitations, particularly the absence of a pain-based inclusion criterion, as participants were not required to have orofacial pain prior to intervention. Despite the null findings in pain reduction, this study reported potential benefits of laser auriculotherapy in other TMD-related symptoms, indicating that its therapeutic effects may extend beyond direct pain relief [18]. The trial’s methodological quality was evaluated as ‘poor’ (PEDro 2/10). Although it offers exploratory data regarding secondary symptoms, the absence of fundamental bias-control measures, such as random allocation, concealed allocation, and blinding, reveals significant methodological fragility that restricts the internal validity of the reported outcomes.

Statistical significance has been demonstrated for the treatment of chronic lower back pain in randomized clinical trials. Menezes et al. evaluated photobiomodulation in auriculotherapy as a monotherapy in a randomized clinical trial involving 50 adults (aged 18–65 years) with chronic spinal pain (duration ≥ 3 months, Numeric Pain Rating Scale [NPRS] > 4). Using two-way repeated-measures ANOVA and Sidak’s post-hoc test, the study reported statistically significant reductions in chronic pain intensity (p < 0.001) at all assessment points, as well as a significant increase in pain threshold [19]. Moreover, a clinically meaningful improvement in pain intensity was observed, consistent with the recommendations of the Initiative on Methods, Measurement, and Assessment of Pain in Clinical Trials (IMMPACT) [19]. The intervention resulted in a substantial reduction in pain, as measured by the Numeric Pain Rating Scale, with a 65.20% decrease from baseline to post-intervention and a 58.62% decrease from baseline to the 15-day follow-up [19]. Regarding methodological quality, the trial achieved a score of 6/10, which is classified as ‘good’ quality. While this reflects a fair degree of rigor, the absence of concealed allocation and participant blinding indicates potential biases that must be considered when interpreting these clinical outcomes.

Similar findings were reported by Mantuani et al., who evaluated the combined use of laser auriculotherapy and cupping therapy [20]. In this randomized clinical trial, 50 adults (aged 18–59 years) with chronic spinal pain (duration ≥ 3 months, Numerical Pain Scale [NVPS] ≥ 3) were divided into an experimental group (n = 25) and a control group (n = 25). Using repeated measures ANOVA and Sidak’s post hoc test, the study demonstrated a significant reduction in pain intensity scores at all assessed time points (p < 0.0001), an increase in pain threshold (p = 0.023), and a clinical improvement, also consistent with IMMPACT guidelines, characterized by a 53% reduction in pain both between pre- and post-intervention and between pre-intervention and the 15-day follow-up [20]. An appraisal of the study’s internal validity resulted in a PEDro score of 7/10, which is classified as ‘good’ quality. The study demonstrates considerable methodological rigor, particularly regarding random allocation and baseline comparability; however, the lack of therapist blinding and intention-to-treat analysis remains a limitation that should be considered when interpreting the robustness of these findings.

Among the studies included in this review, two were identified as feasibility studies, which are designed to test protocol viability rather than to confirm clinical efficacy. The first consisted of a double-blind, randomized, placebo-controlled clinical trial [21] for the treatment of knee osteoarthritis, which evaluated the use of laser auriculotherapy and magnetic tablets among four groups: (1) laser therapy plus magnetic tablets, (2) placebo laser therapy plus magnetic tablets, (3) laser therapy plus placebo tablets and (4) placebo laser therapy plus placebo tablets. The study recruited 60 adults aged 60 years or older diagnosed with knee osteoarthritis based on the American College of Rheumatology clinical criteria. Using Kruskal–Wallis tests for between-group comparisons and Wilcoxon sign-ranked tests for within-group assessments with a significance level of p < 0.05, the researchers found no statistically significant difference in the pain scale after therapy among the groups. However, there were significant relative differences according to the Cohen effect [22], with Cohen’s f of 0.319 (intermediate effect), in the pain scale values among the intra group participants, with pain reductions of µ = −2.40 for laser alone and µ = −2.54 for the combined treatment. The researchers concluded that the adopted protocol is viable for elderly people with knee osteoarthritis and can be replicated in studies with larger samples [21]. Regarding methodological quality, the trial achieved a PEDro score of 7/10, which is classified as ‘good’ quality. The study demonstrates significant methodological rigor, particularly through the use of placebo-controlled blinding; however, the absence of intention-to-treat analysis represents a methodological vulnerability that should be acknowledged when considering the reliability of these preliminary findings.

The second feasibility study, conducted by Marques et al., was a double-blind clinical trial investigating the treatment of temporomandibular dysfunction (TMD) using photobiomodulation applied to acupuncture auricular points [23]. This study included women aged 18 to 60 years with a clinical diagnosis of TMD confirmed by the RDC/TMD protocol. Using SPSS 20.0, the researchers applied the Mann-Whitney test to compare quantitative dependent variables between groups and the Wilcoxon test for intragroup temporal comparisons, with a significance level of p < 0.05. Participants in the control group received the same established treatment protocol; however, the photobiomodulation device was kept turned off. The study found a statistically significant difference between the groups in terms of pain intensity, specifically, immediate pain (p = 0,04) and the worst facial pain reported (p = 0,01) [23]. The authors concluded that the study design is feasible. They estimated that a sample size of 11 participants per group would be sufficient to assess pain intensity using the Graded Chronic Pain Scale, which served as the primary outcome. For secondary outcomes, 25 participants would be needed to assess symptoms of generalized anxiety (using the Generalized Anxiety Disorder scale), and 22 participants for depression assessment (using the Patient Health Questionnaire) [23]. Regarding methodological quality, the trial achieved a PEDro score of 9/10, which is classified as ‘excellent’ quality. This score reflects a high degree of methodological rigor, including effective randomization, concealed allocation, and blinding; however, the absence of intention-to-treat analysis remains a minor limitation that should be noted when evaluating the robustness of the study’s conclusions.

#### Acute postoperative pain

A randomized clinical trial by Sampaio Filho et al. evaluated the effectiveness of PBM at acupuncture auricular points compared to a placebo (inactive laser) for acute postoperative pain relief following tooth extraction [24]. The study included 38 healthy young adults (aged 18–28 years) who underwent 76 bilateral surgeries. To analyze pain and secondary outcomes (mouth opening, edema, and local temperature), the authors employed mixed-effects models including random effects at the patient level and treated data as an unbalanced panel, with sex, medication use, and surgery duration as covariates. The analysis, performed with STATA 15 software at a 0.05 significance level, revealed no significant differences in pain levels between the groups at baseline, 24 h, 48 h, or 7 days post-surgery (all *p* > 0.05). Additionally, the overall mean pain levels did not differ significantly between groups [24]. In terms of methodological quality, the trial attained a score of 7/10, categorized as ‘good’. While the study exhibits high levels of rigor through proper random allocation, concealed allocation, and blinded assessment, the lack of participant blinding and intention-to-treat analysis constitutes a limitation that warrants careful consideration.

A synthesis of the studies included in this review reveals a heterogeneous body of evidence regarding the efficacy of laser auriculotherapy for pain management. With an average PEDro score of 6.14, the research ranges from poor to excellent in methodological quality. While several trials demonstrate promising results and highlight the viability of therapeutic protocols, the inconsistency in bias control measures, particularly the frequent absence of therapist blinding and intention to treat analysis, underscores the need for more standardized, high rigor clinical investigations to definitively establish the clinical impact of this intervention.

As a safety outcome, none of the included studies reported adverse effects, skin reactions, or local discomfort associated with the auricular laser interventions.

### Laser parameters

As summarized in Table 2, the included studies presented variability in the operational parameters and technical specifications of the lasers used. Four studies employed low-power infrared lasers with wavelengths 808–904 nm [17–20], while three studies used red diode lasers (650–660 nm) [24–21, 23]. Reported energy densities varied from 0.54 to 4 J/cm², while applied energies reached up to 4 J. Only a few studies provided additional details, such as emission area, pulse frequency, or power density, highlighting the heterogeneity and incomplete reporting of laser parameters across protocols.

**Table 2:** Definition of operational parameters and technical specifications of the lasers

	Laser type	Output power or average power	Wavelength	Energy density	Power density	Applied energy	Other technical parameters
Fernandes MP (2020)	Low-intensity infrared laser (Therapy EC Duo Mom)	100 mW	808 nm	Not reported	Not reported	Not reported	Not reported
Rodriguez, MF (2019)	*Endophoton − 75 W pulsed diode laser (InGaAs\GaAs).*	50 mW	904 nm	4 J/cm²	Not reported	Not reported	Emission area of 0.01 cm²; Pulse width of 100 ns.
Menezes F da S (2022)	Low power infrared laser (Therapy EC - DMC^®^)	100 mW	808 nm	Not reported	Not reported	4 J	Not reported
Mantuani (2024)	Low-power infrared laser (Therapy EC) from DMC^®^.	100 mW	808 nm	Not reported	Not reported	4 J	Not reported
Sampaio-Filho H (2018)	Red diode laser (Therapy XT ^®^ DMC).	100 mW	660 nm	Not reported	35.4 mW/cm²	1 J	Emission area of 0.002826 cm²
Suen LK (2016)	Pointer Pulse Laser	2.5 mW	650 nm	0.54 J/cm²	Not reported	Not reported	10 Hz pulse
Marques SL (2023)	Acupuncture – Ecco Optical Fibers and LTDA (Pulsed Laser)	100 mW	660 nm	4 J/cm²	100 mW/cm²	4 J	Emission area of 1.0 cm²; 9.12 Hz pulse

### Treatment protocolos

As detailed in Table 3, all studies applied auricular laser stimulation targeting classical auricular acupuncture points, most commonly Shen Men (*n* = 6; 85.7%), Sympathetic (*n* = 3; 42.8%), Kidney (*n* = 3; 42.8%), Liver (*n* = 3; 42.8%), and Subcortex (*n* = 3; 42.8%). The number of treatment sessions ranged from 3 to 18, with most protocols performing 8 to 10 sessions, one to two times per week. Two trials combined auricular laser with additional therapies, such as systemic cupping therapy [20] and magnetic tablets (MAT) [21]. Control conditions varied across trials and included no intervention [18–19, 20], placebo laser (device off) [23, 24], or an active comparator such as occlusal splint therapy [17]. The included trials comprised small-to-moderate sample sizes and varied follow-up durations, along with a notable predominance of female participants across the studies.

**Table 3:** Study characteristics, participant demographics, and intervention protocols

	Sample Size, age and gender	Selected auricular points	Number of sessions, Frequency & Follow-up	Combined techniques	Control group
Fernandes MP (2020)	N = 40, dropout: 5%. Laser auriculotherapy group n = 19, mean age 35.4 ± 11.62 y; Control group n = 19, mean age 24.4 ± 2.26 y. Female: 85.4%	Shen men, Upper Limb, Zero Point, Stomach, Maxilla, Mandible, Anxiety and Stress.	10 sessions, 1x per week. Evaluated at pre-intervention, post-intervention, and post-treatment evaluation.	No	The control group received no intervention.
Rodriguez, MF (2019)	N = 40, dropout: 47.5%. Occlusal splint group n = 11, mean age 43.63 y; Laser auriculotherapy group n = 10, mean age 47.5 y. Female: 100%	Shen Men, Heart and Temporomandibular Joint (TMJ).	8 sessions, 1x per week. Evaluated at pre-intervention, post-intervention, and follow-ups at 4 weeks and 8 weeks.	No	Occlusal Splint
Menezes F da S (2022).	N = 50, dropout: 6%. Laser auriculotherapy group n = 24, mean age 48.92 ± 15.22 y; Control group n = 23, mean age 40.83 ± 10.70 y. Female: 70.21%	Shen men (TF4), Kidney (CO10), Sympathetic nerve (AH6a), Bladder (CO9), Liver (CO12), Subcortex (AT1), and Point corresponding to the location of pain (cervical vertebrae (AH13), thoracic vertebrae (AH11) and lumbosacral vertebrae (AH9)).	10 sessions, 2 x per week. Evaluated at pre-intervention, post-intervention, and a 15-day follow-up.	No	The control group received no intervention.
Mantuani (2024)	N = 50, dropout: 6%. Laser auriculotherapy group n = 24, mean age 35.54 ± 2.76 y; Control group n = 23, mean age 40.83 ± 2.28 y. Female: 63.8%	Shen men (TF4), Kidney (CO10), and Sympathetic (AH6a), Liver (CO12), Subcortex (AT1), Bladder (CO9) and Point corresponding to the location of pain (cervical vertebrae (AH13), thoracic vertebrae (AH11) and lumbosacral (AH9)).	10 sessions, 2 x per week. Evaluated at pre-intervention, post-intervention and 15 days post-treatment (follow-up)	Systemic cupping therapy in acupuncture points (5 sessions, 1x per week).	The control group received no intervention.
Sampaio Filho (2018)	*N* = 42, dropout: 9.52%. Mean age 24.5 ± 4.3 y. Female: 69.05%	Shen Men, Sympathetic (SNV), Stomach, Toothache, Jaw, Adrenal.	3 sessions, immediately post-op, and at 24 and 48 h after surgery. Evaluated at baseline, 24 h, 48 h, and 7 days.	No	Placebo laser auricular (Laser off).
Suen LK (2016)	N = 44, dropout: 2.27%. Mean age 72.66 ± 7.04 y. Female: 95.35%)	Shen Men (TF4), Knee (AH4), Spleen (CO13), Liver (CO12), Kidney (CO10) and Subcortex (AT4).	18 sessions, 3 x per week. Evaluated at pre-intervention, post-intervention, and post-treatment evaluation.	Magnetic tablets (MAT) − 200 Gauss/1.76 mm diameter;	Placebo Laser Auricular (Placebo LAT; Laser off) + MAT; Laser Auricular + Placebo MAT (Plaster tablets). Placebo LAT + Placebo MAT.
Marques SL (2023)	N = 30, dropout: 30.0%. Laser auriculotherapy group n = 10, mean age 35.62 ± 7.70 y; Control group n = 11, mean age 41.5 ± 12.54 y. Female: 100%	Zero Point, Emotional Scar Point, Cervical Rib, ACTH, TMJ (French Auricular Acupuncture Points).	4 sessions, 1x per week. Evaluated at pre-intervention and post-intervention (after 4 sessions).	No	Placebo laser auricular (Laser off).

## Discussion

### Evidence of effectiveness and results

The studies reviewed indicate that photobiomodulation in auriculotherapy has shown promising results in reducing pain, particularly in chronic spinal conditions [19, 20] and in orofacial pain related to TMD [17]. These findings highlight its potential as a promising, noninvasive approach for managing chronic musculoskeletal pain. Only two studies did not demonstrate significant analgesic effects, one addressing postoperative pain after third molar extraction [24] and another evaluating orofacial pain in TMD [18]. Overall, the evidence supports laser auriculotherapy as an emerging modality with encouraging applications in pain management.

Furthermore, two feasibility studies, one conducted with patients with knee osteoarthritis [21] and another conducted with patients with TMD [23], which indicated that protocols with laser auriculotherapy are viable and promising, and can be applied in future clinical studies with larger samples to evaluate analgesic efficacy.

#### Chronic pain conditions vs. acute postoperative pain

The differences in the studies that used PBM treatment for painful TMD [17–18] demonstrate important divergences that can be attributed to the methodological criteria adopted, mainly related to the selection of participants. Fernandes et al. presented a notable methodological limitation regarding the lack of standardization in requiring participants to present with orofacial pain at the time of selection [18]. Therefore, it is important to adopt established guidelines for clinical trials, such as the IMMPACT recommendations, which advise that when pain is evaluated as a primary outcome, patient selection should require a minimum pain intensity score of of ≥ 4 on a validated scale [25]. This threshold ensures that participants present with sufficient pain levels to allow for a meaningful observation of treatment-related improvements [25]. Rodrigues et al. adopted stricter inclusion criteria, requiring participants to present with orofacial pain confirmed by both a numerical scale and dynamometer-based palpation [17].

These methodological discrepancies are further underscored by their respective PEDro scores. While Rodrigues et al. [17] achieved a score of 5/10, Fernandes et al. [18] obtained a score of 2/10. This lower rating for Fernandes et al. stems from a substantial deficit in the implementation of randomized controlled trial standards, including inadequate participant selection and a lack of experimental controls, which compromises data reliability and limits the clinical applicability of their conclusions.

In contrast, the feasibility study conducted by Marques et al. [23] exemplifies a robust experimental design investigating TMD. With an excellent classification of PEDro 9/10, this trial addressed the gaps in blinding and the assignment process found in previous investigations [17, 18], strengthening internal validity through validated diagnostic criteria (RDC/TMD) and evidence-based sample size calculations. The emphasis on methodological rigor is further supported by the meta-analysis of Mira et al. [9] which confirms that when protocols are standardized, photobiomodulation at acupuncture points effectively attenuates spontaneous pain with high consistency. Conversely, the high heterogeneity observed in broader analyses reinforces that the precise methodological standardization established by Marques et al. [23] is critical for achieving predictable clinical outcomes. Consequently, the advancement of laser auriculotherapy for TMD management is contingent upon replicating this rigorous standard to generate the high-quality evidence required to define definitive therapeutic parameters.

The studies that evaluated the treatment of chronic low back pain presented statistically and clinically significant results in reducing pain levels and increasing the nociceptive threshold. In this sense, the results demonstrated the effectiveness of this therapeutic modality, as they also used the same laser device, laser parameters, and auriculotherapy protocol (auricular points, number of sessions, and treatment time) [19, 20]. However, with PEDro scores of 6/10 (good) [19] and 7/10 (good) [20], these findings must be interpreted with caution; the lack of concealed allocation and participant blinding introduces potential bias that tempers the robustness of the evidence. Both studies showed effectiveness, but it would be important to carry out a comparative analysis between the data to observe whether the combination of techniques generated more consistent and lasting results than the isolated use of PBM.

These data reinforce the clinical applicability of PBM in chronic musculoskeletal pain conditions, highlighting its potential to reduce perceived pain and modify patients’ nociceptive sensitivity. Furthermore, the positive effects observed with laser auriculotherapy in the treatment of chronic spinal pain are corroborated by other studies in the literature that used conventional auriculotherapy as a treatment, which also reported relevant clinical benefits [3, 26–27]. Ultimately, these results underscore the need for more stringently controlled trials to definitively establish therapeutic efficacy.

Regarding the potential for laser auriculotherapy to address chronic pain, transcutaneous auricular vagus nerve stimulation (taVNS) serves as a relevant reference. Evidence indicates that taVNS modulates central pain processing by stimulating peripheral auricular vagal nerve endings [28]. This process is understood to trigger descending inhibitory pathways, frequently involving the periaqueductal gray and the rostral ventromedial medulla, and to modulate inflammatory pathways, including the cholinergic anti-inflammatory reflex [28]. Laser auriculotherapy targets these same peripheral auricular endings, raising the possibility that it may operate through similar neuromodulatory mechanisms, provided that optical parameters are calibrated to reach the required signaling threshold. However, these hypotheses warrant rigorous validation through future research. Preclinical experimental studies in animal models are essential to elucidate the precise neurobiological signaling pathways involved, while functional magnetic resonance imaging (fMRI) investigations could provide critical evidence of brain activation patterns in humans, thereby confirming whether laser auriculotherapy effectively engages these central pain-processing regions.

The double-blind, randomized, placebo-controlled trial by Suen, Yeh, and Yeung evaluated laser in auriculotherapy for knee osteoarthritis [21]. Although the study did not reach statistical significance in between-group comparisons, it observed clinically relevant improvements, suggesting a therapeutic benefit. Regarding methodological quality, the trial achieved a PEDro score of 7/10 (good); however, the absence of intention-to-treat analysis remains a vulnerability to be acknowledged. Furthermore, it is possible that the low energy density used in the dosimetric protocol limited the clinical effect, an aspect that will be discussed in further detail. This limitation underscores that the clinical efficacy of optical neuromodulation is inherently dependent on the total energy delivered and the biological dose, which must be precisely calibrated to effectively engage peripheral and central signaling pathways. While these results differ from the significant pain reduction reported by Zhang et al., who investigated knee osteoarthritis using conventional auriculotherapy [29], the intermediate effect sizes observed in Suen et al. [21] suggest that the intervention still holds therapeutic potential. These findings underscore that future research must incorporate a refined selection of technical parameters to further investigate the efficacy of laser auriculotherapy in managing advanced osteoarticular conditions.

In the study conducted by Sampaio Filho et al., no significant differences were observed between groups receiving photobiomodulation in auriculotherapy for acute postoperative pain after third molar extraction [24]. This trial achieved a PEDro score of 7/10 (good), indicating substantial methodological rigor despite the null findings. This result may also be associated with the specific dosimetric protocol used, as discussed below. In contrast, Vaira et al., in a split-mouth, randomized, placebo-controlled study evaluating the same surgical procedure using conventional auriculotherapy with vaccaria seeds, reported a measurable analgesic effect [30]. The continuous physical presence of vaccaria seeds, which allows for ongoing manual pressure over several days, provides the uninterrupted somatic stimulus required to overcome immediate postoperative nociception. This importance of sustained stimulation is corroborated by Yeh et al. [31], who highlight that for acute conditions characterized by immediate tissue trauma, the brief window of an intermittent laser protocol restricted to the session time may not reach the neural signaling threshold necessary to suppress the rapid post-surgical cascade of pro-inflammatory cytokines. Consequently, the challenge for laser auriculotherapy in acute pain settings is to reconcile its intermittent application with the sustained neural signaling required to match the continuous efficacy of manual modalities. Future research should investigate whether optimizing application frequency, dose or intervals can bridge this gap, ensuring that laser parameters are dynamically adapted to the rapid progression of the post-surgical inflammatory response.

Despite the positive effects and especially the low cost of conventional auriculotherapy with seeds and needles, its use depends on patient acceptance and tolerability. Reported adverse events with seeds include local skin irritation, tenderness or pain at the taped site, mild to moderate discomfort, dizziness, ear skin breakdown, and pressure ulcers in the auricular pavilion. Auriculotherapy with needles also carries additional risks of local infection, inflammation at the puncture site and bleeding because of the particular characteristics of the auricular pavilion [32–33]. By contrast, laser acupuncture (LA) is a noninvasive, atraumatic technique that avoids these complications, being generally painless, with only mild transient side effects such as dizziness, headache, or fatigue [6, 34–35], and offers additional advantages including shorter treatment duration, easier simulation of placebo conditions and safe acupoint stimulation [6].

### Laser parameters

Currently, there are no standardized guidelines for the technical parameters of PBM applied to auriculotherapy and systemic acupuncture. This gap hinders the replicability of studies and contributes to the inconsistency of the results obtained [35]. In general, the therapeutic effects of PBM depend directly on specific technical variables. The lasers used can be continuous, which emit light constantly, or pulsed, which emit light at regular intervals [36]. This distinction interferes with the way in which the energy interacts with the tissues. Furthermore, the World Association for Laser Therapy (WALT) recommends that researchers provide a complete description of the parameters and corresponding units used in low-level laser therapy (LLLT). These include the application procedure, wavelength (nm), average laser output (mW), treatment time (s), energy dose (J), energy density (J/cm²), power density (mW/cm²), spot size (cm²) and total energy delivered across all sessions (J) [37].

The parameters of low-density lasers applied to auricular and acupuncture points are comparable, with the following ranges: power output (0.001–0.1 W), wavelength (300–10,600 nm), pulse rate (continuous to 5,000 Hz), pulse duration (1–500 ms), interpulse interval (1–500 ms), power density (0.01–10 W/cm²), and dose (power × irradiation time/area) of 0.01–100 J/cm² [38–39]. Although pulse duration characterizes pulsed laser delivery, only one of the three studies employing pulsed lasers reported this information (100 ns) [17].

Discrepancies in laser equipment and configuration create further challenges in standardizing treatment parameters. While the literature typically reports energy density (J/cm²) as the key biomodulatory parameter, many modern devices allow the operator to select only the total energy in joules, which alters the energy density depending on the emission area. Across the studies reviewed, the laser emission area varied considerably: 0.002826 cm² [24], 0.01 cm² [17], and 1.0 cm² [23].

According to the Arndt-Schultz curve, very low energies do not produce a therapeutic effect, while excessive doses can inhibit cellular functions, with the effective therapeutic range generally between 1 and 4 J/cm² [40]. Among the reviewed studies, energy densities varied: 4 J/cm² [17, 23], and 0.54 J/cm² [21]. Only one study clearly reported both the total energy applied per point (4 J) and the corresponding energy density (4 J/cm²) [23]. In Sampaio Filho et al., reported the total energy applied per point (1 J) and the energy density could theoretically be calculated, but the resulting value (~ 354 J/cm²) was far above typical ranges, indicating discrepancies between reported and calculated parameters. In studies that reported only the total delivered energy [19, 20], it was not possible to determine the energy density (fluence), which represents the effective treatment dose [6, 41]. For example, both studies reported a total energy of 4 J; however, without specifying the emission area, the corresponding energy density (J/cm²) could not be calculated, thereby limiting comparability with other studies and compromising treatment reproducibility.

The Australian Medical Acupuncture College recommends an energy density of 4 J/cm² as ideal for laser biostimulation at acupuncture points [41–42]. However, some authors emphasize that the dose should be adjusted according to the individual response of each patient [41].

In addition to the variations in dose, there were discrepancies in wavelengths. In Litscher & Opitz, the range from 650 to 900 nm is considered as the ideal range for tissue penetration [41]. Taking this first range into account, only one study used a slightly longer length (904 nm) [17]. However, in Hamblin [43], the range of 690 nm and 860 nm is considered for ideal tissue penetration. For this second range, only three studies are within this range using the wavelength of 808 nm [18–19, 20]. Establishing an ideal wavelength is not yet possible, and perhaps this may be unlikely since the variety of wavelengths has its advantages depending on the purpose of its use in the different stimulated tissues [43].

Furthermore, this review identified considerable variation and frequent inadequate reporting of laser parameters. A critical gap is that many trials fail to state essential dosimetric data, such as energy density and spot size. This lack of transparency prevents a clear synthesis of evidence. This limitation was also noted in previous reviews on laser acupuncture for temporomandibular disorders [9], myofascial pain [10], and chronic low back pain [11]. Such discrepancies in technical parameters, such as laser type, dose, frequency, and irradiated area, highlight a lack of methodological standardization which hinders comparative analysis, undermines accurate evaluation of photobiomodulation effects, and limits the development of evidence-based therapeutic recommendations [35].

### Treatment protocols

Due to the different nomenclatures in Chinese and French auriculotherapy, it is important that studies report both the name and exact location of the points used for subsequent evaluation [6]. It should also be noted that differences exist in the points, their locations, and their functions depending on the branch of auriculotherapy applied, whether Chinese or French (Western) [44].

It is notable that all studies of this review provided the exact location of the auriculotherapy points used. In addition, three studies [19–21] reported the point location method according to the World Federation of Acupuncture and Moxibustion Societies (WFAS), which is a Chinese auricular acupuncture standardization method that is considered an international standard and recognized by the World Health Organization (WHO), being seen as the most appropriate in the distribution and location of points [45, 46].

The Shen Men auricular point was used in almost all studies, and this finding was similar in other review studies that evaluated conventional auriculotherapy in the treatment of pain [2, 47–49]. Selecting precise points, such as Shen Men, reflects an attempt at neuroanatomical accuracy, as distinct auricular regions possess unique neurovascular profiles [4, 48]. This approach ensures that the application is spatially optimized, even before considering the specific photonic parameters.

Regarding the number of sessions performed, there were variations between studies, with a minimum number of sessions performed being 3 sessions [24] which studied acute pain and the maximum, 18 sessions [21] which studied chronic pain. This finding is similar to what was revised by Morais et al. in which no pattern was obtained in the number of auriculotherapy sessions offered in trials for the treatment of musculoskeletal pain [50].

This variability in selected auricular points, treatment frequency underscores the lack of protocol standardization among studies investigating laser auriculotherapy in the pain treatment. When interpreted through PBM principles, these parameters act as critical regulators of the biological response rather than mere procedural choices. In accordance with the Arndt-Schultz law [40], establishing an optimal application frequency is essential to achieve the cumulative energy threshold required for mitochondrial stimulation without inducing cellular accommodation or inhibitory overdosage. Therefore, future research must shift from purely descriptive protocol reporting to the rigorous definition of energy densities and application intervals. Standardizing these temporal and technical parameters is fundamental not only to ensure clinical reproducibility but also to allow for the systematic evaluation of how PBM synergizes with auricular neural structures to achieve therapeutic goals.

### Limitation of the study

Some limitations of this scoping review should be noted. Although the search strategy was comprehensive, the number of included studies was small, highlighting a general scarcity of literature combining laser and auriculotherapy for pain management. Additionally, regional databases from China, Korea, and Japan were not searched, which may have resulted in the omission of relevant papers, especially considering the traditional roots of auriculotherapy. While no language restrictions were applied, the utilized databases primarily index publications in English. Significant heterogeneity was observed in laser dosimetry and clinical protocols across the included studies. Furthermore, methodological appraisal using the PEDro scale revealed consistent vulnerabilities in bias control, specifically the frequent absence of intention-to-treat analysis and therapist blinding. These methodological inconsistencies complicate direct comparisons and limit the internal validity of the reported outcomes. Finally, in accordance with scoping review frameworks, the findings are restricted to a narrative synthesis, providing an overview of the evidence quality and methodological rigor rather than a quantitative meta-analysis. 

## Conclusion

This scoping review allowed the mapping of studies regarding the use of lasers in auriculotherapy. The amount of research found shows that this is a topic that has been little studied. Another relevant aspect is the presence of weaknesses in the reporting and conduct of clinical trials, which need to present more details to ensure the quality of the evidence. In addition, the health conditions treated and the approaches identified vary between studies, with discrepancies between the types of lasers and their parameters and many gaps in information about them, making comparison between results complex.

Despite this, the results of the studies indicate that the analgesic effect of laser photobiomodulation in auriculotherapy, whether used alone or with another method, was considered positive and brought benefits in most studies. These findings suggest a promising clinical potential, indicating that auricular laser therapy may contribute to the management of chronic musculoskeletal pain. It is therefore recommended that new studies be conducted to evaluate the effects of laser use in auriculotherapy, with particular attention to the clear reporting of technical parameters, safety outcomes and adverse events. Such efforts are essential to improve reproducibility and to support the development of future guidelines in laser auriculotherapy. 

## Supplementary Information

Below is the link to the electronic supplementary material.

Supplementary Material 1

Supplementary Material 2

Supplementary Material 3

## Data Availability

No datasets were generated or analysed during the current study.

## References

[1] - Hou PW, Hsu HC, Lu TS et al (2015) The history, mechanism, and clinical application of auricular therapy in traditional Chinese medicine. Evid Based Complement Alternat Med 2015:495684. 10.1155/2015/495684 26823672 PMC4707384

[2] - Asher GN, Jonas DE, Coeytaux RR et al (2010) Auriculotherapy for pain management: a systematic review and meta-analysis of randomized controlled trials. J Altern Complement Med 16(10):1097–1108. 10.1089/acm.2009.0451 20954963 PMC3110838

[3] - Yang LH, Wang XH, Chen Y et al (2017) Efficacy of auricular acupressure for chronic low back pain: a systematic review and meta-analysis of randomized controlled trials. Evid Based Complement Alternat Med 2017:638364. 10.1155/2017/6383649 PMC5539928 28804504

[4] - Butt MF, Albusoda A, Farmer AD, Aziz Q (2019) The anatomical basis for transcutaneous auricular vagus nerve stimulation. J Anat 236(4):588–611. 10.1111/joa.13122 31742681 PMC7083568

[5] - Peuker ET, Filler TJ (2002) The nerve supply of the human auricle. Clin Anat 15(1):35–37. 10.1002/ca.1089 11835542

[6] - Round R, Fernandes MP, Prado MC et al (2013) Auricular acupuncture with laser. Evid aruBased Complement Alternat Med 2013:984763. 10.1155/2013/984763 PMC3710613 23935695

[7] - Freitas LF, Hamblin MR (2016) Proposed mechanisms of photobiomodulation or low-level light therapy. IEEE J Sel Top Quantum Electron 22(3):348–364. 10.1109/JSTQE.2016.2561201 PMC5215870 28070154

[8] - Dompe C, Moncrieff L, Matys J et al (2020) Photobiomodulation—underlying mechanism and clinical applications. J Clin Med 9(6):1724. 10.3390/jcm9061724 32503238 PMC7356229

[9] - Mira PCS, Silva AC, Oliveira RF et al (2024) Laser acupuncture to reduce temporomandibular disorder (TMD) symptoms: systematic review and meta-analysis. Lasers Med Sci 39(1):1. 10.1007/s10103-024-03999-z 38374226

[10] - Law D, McDonough S, Bleakley C, Baxter GD, Tumilty S (2015) Laser acupuncture for treating musculoskeletal pain: a systematic review with meta-analysis. J Acupunct Meridian Stud 8(1):2–16. 10.1016/j.jams.2014.10.004 25660439

[11] - Mao X, He H, Ding J (2024) Efficacy of laser acupuncture for treatment of chronic low back pain: a systematic review and meta-analysis. Pain Manag Nurs 25(5):529–537. 10.1016/j.pmn.2024.05.001 38821755

[12] - Aromataris E, Lockwood C, Porritt K, Pilla B, Jordan Z (eds) (2024) JBI Manual for Evidence Synthesis*.* JBI; Available from: https://synthesismanual.jbi.global.

[13] - Ouzzani M, Hammady H, Fedorowicz Z, Elmagarmid A (2016) Rayyan—a web and mobile app for systematic reviews. Syst Rev 5:210. 10.1186/s13643-016-0384-4 27919275 PMC5139140

[14] - Tricco AC, Lillie E, Zarin W et al (2018) PRISMA Extension for Scoping Reviews (PRISMA-ScR): checklist and explanation. Ann Intern Med 169:467–473. 10.7326/M18-0850 30178033

[15] - Cashin AG, McAuley JH (2020) Clinimetrics: Physiotherapy Evidence Database (PEDro) Scale. J Physiother 66(1):59. 10.1016/j.jphys.2019.08.005 31521549

[16] - Page MJ, McKenzie JE, Bossuyt PM et al (2021) The PRISMA 2020 statement: an updated guideline for reporting systematic reviews. BMJ 372:n71. 10.1136/bmj.n71 33782057 PMC8005924

[17] - Rodrigues MF, Fernandes MP, Prado MC et al (2019) Effects of low-power laser auriculotherapy on the physical and emotional aspects in patients with temporomandibular disorders: a blind, randomized, controlled clinical trial. Complement Ther Med 42:340–346. 10.1016/j.ctim.2018.12.010 30670264

[18] - Fernandes MP, dos Santos ALC, Prado MC et al (2020) Effects of photobiomodulation on auriculotherapy points for sleep disorders, anxiety, and temporomandibular dysfunctions. Cranio 41(4):362–367. 10.1080/08869634.2020.1853896 33287687

[19] - Menezes FDS, Fernandes MP, Prado MC et al (2022) Effects of low-power laser auriculotherapy on chronic spinal pain: randomized clinical trial. Complement Ther Clin Pract 48:101578. 10.1016/j.ctcp.2022.101578 35405631

[20] - Mantuani APA, Fernandes MP, Prado MC et al (2024) Laser auriculotherapy associated with cupping therapy in chronic spinal pain: randomized controlled clinical trial. J Bodyw Mov Ther 37:194–201. 10.1016/j.jbmt.2023.11.020 38432806

[21] - Suen LKP, Yeh CH, Yeung KW et al (2016) Using auriculotherapy for osteoarthritic knees among elders: a double-blind randomized feasibility study. BMC Complement Altern Med 16:1–9. 10.1186/s12906-016-1242-6 27473749 PMC4966810

[22] - Lenhard W, Lenhard A (2016) Calculation of effect sizes. Psychometrica. 10.13140/RG.2.2.17823.92329

[23] - Marques SL, Fernandes MP, Prado MC et al (2023) The effect of photobiomodulation auriculotherapy in the treatment of temporomandibular disorders: a double-blind randomized feasibility study. J Photochem Photobiol 18:100210. 10.1016/j.jpap.2023.100210

[24] - Sampaio-Filho H, Fernandes MP, Prado MC et al (2018) Low-level laser treatment applied at auriculotherapy points to reduce postoperative pain in third molar surgery: a randomized, controlled, single-blinded study. PLoS ONE 13(6):e0197989. 10.1371/journal.pone.0197989 29920521 PMC6007895

[25] - Dworkin RH, Turk DC, Peirce-Sandner S et al (2012) Considerations for improving assay sensitivity in chronic pain clinical trials: IMMPACT recommendations. Pain 153(6):1148–1158. 10.1016/j.pain.2012.03.003 22494920

[26] - Moura CC, Fernandes MP, Prado MC et al (2018) Action of ear acupuncture in people with chronic pain in the spinal column: a randomized clinical trial. Lat Am J Nurs 26:3050. 10.1590/1518-8345.2678.3050 PMC6136555 30183875

[27] - Moura CC, Fernandes MP, Prado MC et al (2019) Contribution of Chinese and French ear acupuncture for the management of chronic back pain: a randomized controlled trial. J Clin Nurs 28:3796–3806 31237981 10.1111/jocn.14983

[28] - Zhang J, Zhang Y, Zhao J, Sun J, Zhang X (2026) The role of transcutaneous auricular vagus nerve stimulation in chronic pain: from neurobiological mechanisms to clinical applications. Front Pain Res (Lausanne) 7:1733445. 10.3389/fpain.2026.1733445 41694119 PMC12894318

[29] - Zhang X, Li Y, Wang Y et al (2021) Auricular acupressure for treating early stage of knee osteoarthritis: a randomized, sham-controlled prospective study. QJM 115(8):525–529. 10.1093/qjmed/hcab230 34463759

[30] - Vaira LA, Salzano G, De Riu G et al (2023) Efficacy of auriculotherapy in the control of pain, edema, and trismus following surgical extraction of lower third molars: a split-mouth, randomized, placebo-controlled, and triple-blind study. Oral Maxillofac Surg 27:1–10. 10.1007/s10006-023-01140-y 36735078 PMC10914868

[31] - Yeh CH, Lukkahatai N, Huang X et al (2023) Biological correlates of the effects of auricular point acupressure on pain. Pain Manag Nurs 24:19–26. 10.1016/j.pmn.2022.11.004 36543665 PMC9928890

[32] Tan JY, Molassiotis A, Wang T, Suen LKP (2014) Adverse events of auricular therapy: a systematic review. Evid Based Complement Alternat Med 2014:506758. 10.1155/2014/506758 25435890 PMC4241563

[33] - Nielsen A, Gereau RW, Tick H (2020) Risks and safety of extended auricular therapy: a review of reviews and case reports of adverse events. Pain Med 21(6):1276–1293. 10.1093/pm/pnz379 32430505

[34] - Baxter GD, Bleakley C, McDonough S (2008) Clinical effectiveness of laser acupuncture: a systematic review. J Acupunct Meridian Stud 1(2):65–82. 10.1016/S2005-2901(09)60026-1 20633458

[35] - Chon TY, Lee MC, Thompson JM et al (2019) Laser acupuncture: a concise review. Med Acupunct 31(3):164–168. 10.1089/acu.2019.1343 31297170 PMC6604908

[36] - Catorze MG (2009) Laser: fundamentals and indications in dermatology. Med Cutan Iber Lat Am 37(1):5–27

[37] - World Association of Laser Therapy (WALT) (2006) Consensus agreement on the design and conduct of clinical studies with low-level laser therapy and light therapy for musculoskeletal pain and disorders. Photomed Laser Surg 24(6):761–762. 10.1089/pho.2006.24.761 17199479

[38] - Posten W, Wrone DA, Dover JS et al (2006) Low-level laser therapy for wound healing: mechanism and efficacy. Dermatol Surg 31(3):334–340. 10.1111/j.1524-4725.2005.31086 15841638

[39] - Farivar S, Malekshahabi T, Shiari R (2014) Biological effects of low-level laser therapy. J Lasers Med Sci 5(2):58–62 25653800 PMC4291815

[40] - Sommer AP, Pinheiro ALB, Mester AR et al (2001) Biostimulatory windows in low-intensity laser activation: lasers, scanners, and NASA’s light-emitting diode array system. J Clin Laser Med Surg 19(1):29–33. 10.1089/104454701750066910 11547815

[41] - Litscher G, Opitz G (2012) Technical parameters for laser acupuncture to elicit peripheral and central effects: state-of-the-art and short guidelines based on results from the Medical University of Graz, the German Academy of Acupuncture, and the scientific literature. Evid Based Complement Alternat Med 2012:697096. 10.1155/2012/697096 22619693 PMC3350989

[42] - Australian Medical Acupuncture College. Position statement on laser acupuncture (2020) Available from: http://www.chiro.org/acupuncture/FULL/Position_Statement_on_Laser_Acupuncture.shtml

[43] - Hamblin MR, de Sousa MVP, Agrawal T (2016) Handbook of Low-Level Laser Therapy. Pan Stanford, New York, NY

[44] - Oleson T (2014) Auriculotherapy Manual: Chinese and Western Systems of Ear Acupuncture, 4th edn. Churchill Livingstone, Edinburgh

[45] - Neves ML (2023) Auricular Acupuncture and Neuromodulation: Traditional and Contemporary Aspects. Merithus, Florianópolis, pp 49–51

[46] - WFAS, The World Federation of Acupuncture-Moxibustion Societies (2013) Acupuncture point earpiece (WFAS STANDARD-002: 2012). World J Acupunct Moxibust 23(3):12–21. 10.1016/S1003-5257(13)60055-0

[47] - Monteiro IM, Fernandes MP, Prado MC et al (2023) Effect of auricular acupuncture on cancer pain: a systematic review. Braz J Health Rev 6(1):794–812. 10.34119/bjhrv6n1-061

[48] - Artioli DP, Tavares AL, Bertolini GRF et al (2019) Auriculotherapy: neurophysiology, points to choose, indications, and results on musculoskeletal pain conditions. Braz J Pain 2(4):297–306. 10.5935/2595-0118.20190065

[49] Yeh CH, Suen LKP, Lee PH et al (2014) Efficacy of auricular therapy for pain management: a systematic review and meta-analysis. Evid Based Complement Alternat Med 2014:934670. 10.1155/2014/934670 25165482 PMC4140110

[50] - Morais BX, Fernandes MP, Prado MC et al (2020) Auriculotherapy and reducing chronic musculoskeletal pain: integrative review. Braz J Nurs 73(6):1. 10.1590/0034-7167-2019-0394 33263671

