# Supplementary material for: Laser auriculotherapy for the treatment of pain: a scoping review
Source: Lasers Med Sci. 2026 Sep 14;41(1):229. doi: 10.1007/s10103-026-05000-5 (PMC13574864; doi:10.1007/s10103-026-05000-5)
Supplement: Supplementary file 2 [file 10103_2026_5000_MOESM2_ESM.docx]

**Detailed Search Strategies by Database**

This document presents the complete and specific search strategies utilized for each individual database included in this scoping review. Searches were based on the core strategy combining keywords for pain, photobiomodulation, and auriculotherapy, adapted to comply with each platform's language scope.

- MEDLINE (via PubMed)

Search String: (("Pain" OR Ache OR "Physical Suffering") AND ("Laser Therapy" OR "Low Level Laser Therapy" OR Photobiomodulation OR Phototherapy OR Laser) AND (Auriculotherapy OR "Ear Acupuncture" OR "Auricular Acupuncture"))

- LILACS

Search String: (("Pain" OR Ache OR Dor OR Dolor OR "Physical Suffering") AND ("Laser Therapy" OR "Low Level Laser Therapy" OR "Terapia com Luz de Baixa Intensidade" OR "Terapia a Laser de Baixa Intensidade" OR "Terapia por Luz de Baja Intensidad" OR Photobiomodulation OR Phototherapy OR Fototerapia OR Laser OR "Terapia a Laser" OR Laserterapia OR "Terapia por Láser") AND (Auriculotherapy OR "Ear Acupuncture" OR "Auricular Acupuncture" OR "Acupuntura Auricular" OR Auriculoterapia))

- EMBASE

Search String: (("Pain" OR Ache OR "Physical Suffering") AND ("Laser Therapy" OR "Low Level Laser Therapy" OR Photobiomodulation OR Phototherapy OR Laser) AND (Auriculotherapy OR "Ear Acupuncture" OR "Auricular Acupuncture"))

- SCOPUS

Search String: TITLE-ABS-KEY (("Pain" OR Ache OR "Physical Suffering") AND ("Laser Therapy" OR "Low Level Laser Therapy" OR Photobiomodulation OR Phototherapy OR Laser) AND (Auriculotherapy OR "Ear Acupuncture" OR "Auricular Acupuncture"))

- WEB OF SCIENCE (WOS)

Search String: TS=(("Pain" OR Ache OR "Physical Suffering") AND ("Laser Therapy" OR "Low Level Laser Therapy" OR Photobiomodulation OR Phototherapy OR Laser) AND (Auriculotherapy OR "Ear Acupuncture" OR "Auricular Acupuncture"))

- COCHRANE CENTRAL REGISTER OF CONTROLLED TRIALS (CENTRAL)

Search String: (("Pain" OR Ache OR "Physical Suffering") AND ("Laser Therapy" OR "Low Level Laser Therapy" OR Photobiomodulation OR Phototherapy OR Laser) AND (Auriculotherapy OR "Ear Acupuncture" OR "Auricular Acupuncture"))
