# Supplementary material for: Laser auriculotherapy for the treatment of pain: a scoping review
Source: Lasers Med Sci. 2026 Sep 14;41(1):229. doi: 10.1007/s10103-026-05000-5 (PMC13574864; doi:10.1007/s10103-026-05000-5)
Supplement: Supplementary file 3 [file 10103_2026_5000_MOESM3_ESM.docx]

**PEDro scale of quality for included articles.**

| PEDro Criteria | Fernandes et al., 2020^Ref^ | Mantuani et al., 2024^Ref^ | Marques et al., 2023^Ref^ | Menezes et al., 2022^Ref^ | Sampaio-filho et al., 2018^Ref^ | Suen et al., 2016^Ref^ | Rodrigues et al., 2019^Ref^ | |
| --- | --- | --- | --- | --- | --- | --- | --- | --- |
| Item 1. Eligibility criteria (Not scored) | Ok | Ok | Ok | Ok | Ok | Ok | Ok | |
| Item 2. Random allocation | 0 | 1 | 1 | 1 | 1 | 1 | 1 | |
| Item 3. Concealed allocation | 0 | 1 | 1 | 0 | 1 | 1 | 0 | |
| Item 4. Baseline comparability | 0 | 1 | 1 | 1 | 0 | 1 | 1 | |
| Item 5. Blind subjects | 0 | 0 | 1 | 0 | 1 | 0 | 0 | |
| Item 6. Blind therapists | 0 | 0 | 0 | 0 | 0 | 0 | 0 | |
| Item 7. Blind assessors | 0 | 1 | 1 | 1 | 1 | 1 | 1 | |
| Item 8. Adequate follow-up | 0 | 1 | 1 | 1 | 1 | 1 | 0 | |
| Item 9. Intention-to-treat analysis | 0 | 0 | 1 | 0 | 0 | 0 | 0 | |
| Item 10. Between-group comparisons | 1 | 1 | 1 | 1 | 1 | 1 | 1 | |
| Item 11. Point estimates and variability | 1 | 1 | 1 | 1 | 1 | 1 | 1 | |
| Total score | 2 | 7 | 9 | 6 | 7 | 7 | 5 | |
| Average PEDro Score | 6,14 | | | | | | |  |

PEDro scores of 0-3 are considered ‘poor’, 4-5 ‘fair’, 6-8 ‘good’, and 9-10 ‘excellent’, it is important to note that these classifications have not been validated. Furthermore, for trials evaluating complex interventions (e.g., exercise) a total PEDro score of 8/10 is optimal.

The criteria for classification were based on the guidelines provided by the PEDro database (available at: [https://pedro.org.au/english/summary-of-measurement-properties-of-the-pedro-scale/](https://www.google.com/search?q=https%3A%2F%2Fpedro.org.au%2Fenglish%2Fsummary-of-measurement-properties-of-the-pedro-scale%2F)). For further information regarding the clinimetric properties of the scale, please refer to the following publication:

Cashin AG, McAuley JH. Clinimetrics: Physiotherapy Evidence Database (PEDro) Scale. J Physiother. 2020;66(1):59. doi: 10.1016/j.jphys.2019.08.005.
